# Diaqua­bis­(*N*,*N*-diethyl­nicotinamide-κ*N* ^1^)bis[4-(dimethyl­amino)­benzoato-κ*O*]cobalt(II)

**DOI:** 10.1107/S1600536809030980

**Published:** 2009-08-08

**Authors:** Tuncer Hökelek, Hakan Dal, Barış Tercan, Özgür Aybirdi, Hacali Necefoğlu

**Affiliations:** a Hacettepe University, Department of Physics, 06800 Beytepe, Ankara, Turkey; b Anadolu University, Faculty of Science, Department of Chemistry, 26470 Yenibağlar, Eskişehir, Turkey; c Karabük University, Department of Physics, 78050, Karabük, Turkey; d Kafkas University, Department of Chemistry, 63100 Kars, Turkey

## Abstract

The title Co^II^ complex, [Co(C_9_H_10_NO_2_)_2_(C_10_H_14_N_2_O)_2_(H_2_O)_2_], is centrosymmetric. It contains two dimethyl­amino­benzoate (DMAB) and two diethyl­nicotinamide (DENA) ligands and two water mol­ecules, all of them being monodentate. The four O atoms in the equatorial plane around the Co atom form a slightly distorted square-planar arrangement, while the slightly distorted octa­hedral coordination is completed by the two pyridine N atoms of DENA ligands with the Co—N distance of 2.1519 (11) Å in the axial positions. The Co atom is displaced out of the least-squares plane of the carboxyl­ate group by −0.781 (1) Å. The dihedral angle between the carboxyl­ate group and the adjacent benzene ring is 5.05 (7)°, while the pyridine and benzene rings are oriented at a dihedral angle of 71.48 (5)°. In the crystal structure, inter­molecular O—H⋯O and C—H⋯O hydrogen bonds link the mol­ecules into a three-dimensional network. Two weak C—H⋯π inter­actions are also present.

## Related literature

For general background, see: Bigoli *et al.* (1972 ▶); Krishnamachari (1974 ▶). For related structures, see: Hökelek & Necefoğlu (2007 ▶); Sertçelik *et al.* (2009 ▶).
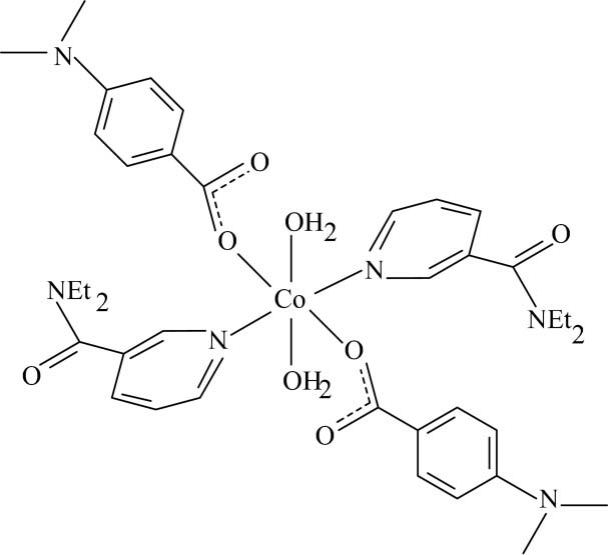


## Experimental

### 

#### Crystal data

[Co(C_9_H_10_NO_2_)_2_(C_10_H_14_N_2_O)_2_(H_2_O)_2_]
*M* *_r_* = 779.79
Monoclinic, 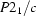

*a* = 6.5184 (1) Å
*b* = 20.4829 (3) Å
*c* = 14.6481 (2) Å
β = 98.492 (1)°
*V* = 1934.31 (5) Å^3^
*Z* = 2
Mo *K*α radiation
μ = 0.50 mm^−1^
*T* = 100 K
0.42 × 0.22 × 0.12 mm

#### Data collection

Bruker Kappa APEXII CCD area-detector diffractometer
Absorption correction: multi-scan (*SADABS*; Bruker, 2005 ▶) *T*_min_ = 0.817, *T*_max_ = 0.942
18749 measured reflections
4821 independent reflections
4137 reflections with *I* > 2σ(*I*)
*R*_int_ = 0.024

#### Refinement

*R*[*F* ^2^ > 2σ(*F* ^2^)] = 0.031
*wR*(*F* ^2^) = 0.081
*S* = 1.05
4821 reflections
251 parameters
3 restraints
H atoms treated by a mixture of independent and constrained refinement
Δρ_max_ = 0.40 e Å^−3^
Δρ_min_ = −0.30 e Å^−3^

### 

Data collection: *APEX2* (Bruker, 2007 ▶); cell refinement: *SAINT* (Bruker, 2007 ▶); data reduction: *SAINT*; program(s) used to solve structure: *SHELXS97* (Sheldrick, 2008 ▶); program(s) used to refine structure: *SHELXL97* (Sheldrick, 2008 ▶); molecular graphics: *Mercury* (Macrae *et al.*, 2006 ▶); software used to prepare material for publication: *WinGX* (Farrugia, 1999 ▶) and *PLATON* (Spek, 2003 ▶).

## Supplementary Material

Crystal structure: contains datablocks I, global. DOI: 10.1107/S1600536809030980/xu2578sup1.cif

Structure factors: contains datablocks I. DOI: 10.1107/S1600536809030980/xu2578Isup2.hkl

Additional supplementary materials:  crystallographic information; 3D view; checkCIF report

## Figures and Tables

**Table 1:** Hydrogen-bond geometry (Å, °)

*D*—H⋯*A*	*D*—H	H⋯*A*	*D*⋯*A*	*D*—H⋯*A*
O4—H41⋯O2^i^	0.908 (16)	1.777 (15)	2.6621 (14)	164.0 (16)
O4—H42⋯O2^ii^	0.907 (16)	1.898 (15)	2.7802 (14)	163.4 (14)
C9—H9⋯O3^iii^	0.95	2.41	3.3447 (17)	168
C19—H19*A*⋯O3^iv^	0.98	2.47	3.403 (2)	160
C15—H15*A*⋯*Cg*2^v^	0.98	2.86	3.734 (2)	148
C18—H18*B*⋯*Cg*1^vi^	0.98	2.86	3.7907 (19)	158

Symmetry codes: (i) 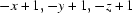; (ii) 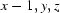; (iii) 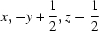; (iv) 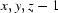; (v) 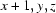; (vi) 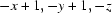. *Cg*1 and *Cg*2 are the cetroids of the C2–C7 and N1/C8–C12 rings, respectively.

**Fig. 1.**
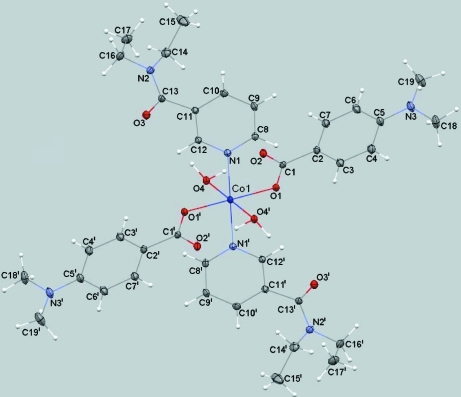
The molecular structure of the title molecule with the atom-numbering scheme. Displacement ellipsoids are drawn at the 50% probability level. Primed atoms are generated by the symmetry operator:(') -x, -y, -z.

## supplementary crystallographic information

### Comment

As a part of our ongoing investigation on transition metal complexes of nicotinamide (NA), one form of niacin (Krishnamachari, 1974), and/or the nicotinic acid derivative *N*,*N*-diethylnicotinamide (DENA), an important respiratory stimulant (Bigoli *et al.*, 1972), the title compound was synthesized and its crystal structure is reported herein.

The title compound is a monomeric complex, with Co^II^ ion on a centre of symmetry, consisting of two DENA and two dimethylaminobenzoate (DMAB) ligands and two water molecules. The structures of similar DENA and/or NA complexes of Co^II^ ion, [Co(C_8_H_5_O_3_)_2_(C_10_H_14_N_2_O)_2_(H_2_O)_2_] (Sertçelik *et al.*, 2009) and [Co(C_9_H_10_NO_2_)_2_(C_6_H_6_N_2_O)_2_(H_2_O)_2_] (Hökelek & Necefoğlu, 2007) have also been determined.

In the title compound, all ligands are monodentate. The four O atoms (O1, O4, and the symmetry-related atoms, O1', O4') in the equatorial plane around the Co atom form a slightly distorted square-planar arrangement, while the slightly distorted octahedral coordination is completed by the two pyridine N atoms (N1, N1') of the DENA ligands at 2.1519 (11) Å from the Co atom in the axial positions (Fig. 1). The average Co—O bond length is 2.0955 (10) Å and the Co atom is displaced out of the least-squares plane of the carboxylate group (O1/C1/O2) by -0.781 (1) Å. The dihedral angle between the planar carboxylate group and the benzene ring A (C2—C7) is 5.05 (7)°, while that between rings A and B (N1/C8—C12) is 71.48 (5)°.

In the crystal structure, intermolecular O—H···O and C—H···O hydrogen bonds (Table 1) link the molecules into a three-dimensional network, in which they may be effective in the stabilization of the structure. Two weak C—H···π interactions (Table 1) are also found.

### Experimental

The title compound was prepared by the reaction of CoSO_4_.7H_2_O (1.41 g, 5 mmol) in H_2_O (50 ml) and DENA (1.78 g, 10 mmol) in H_2_O (50 ml) with sodium *p*-dimethylaminobenzoate (1.88 g, 10 mmol) in H_2_O (100 ml). The mixture was filtered and set aside to crystallize at ambient temperature for one week, giving red single crystals.

### Refinement

Atoms H41 and H42 (for H_2_O) were located in difference Fourier map and refined isotropically, with restrains of O4—H41 = 0.908 (13), O4—H42 = 0.907 (14) Å and H41—O4—H42 = 106.6 (14)°. The remaining H atoms were positioned geometrically with C—H = 0.95, 0.99 and 0.98 Å, for aromatic, methylene and methyl H atoms, respectively, and constrained to ride on their parent atoms, with *U*_iso_(H) = *xU*_eq_(C), where *x* = 1.5 for methyl H and *x* = 1.2 for all other H atoms.

### Crystal data

**Table:**

[Co(C_9_H_10_NO_2_)_2_(C_10_H_14_N_2_O)_2_(H_2_O)_2_]	*F*(000) = 826
*M**_r_* = 779.79	*D*_x_ = 1.339 Mg m^−^^3^
Monoclinic, *P*2_1_/*c*	Mo *K*α radiation, λ = 0.71073 Å
Hall symbol: -P 2ybc	Cell parameters from 9065 reflections
*a* = 6.5184 (1) Å	θ = 2.4–28.3°
*b* = 20.4829 (3) Å	µ = 0.50 mm^−^^1^
*c* = 14.6481 (2) Å	*T* = 100 K
β = 98.492 (1)°	Block, red
*V* = 1934.31 (5) Å^3^	0.42 × 0.22 × 0.12 mm
*Z* = 2	

### Data collection

**Table:**

Bruker Kappa APEXII CCD area-detector diffractometer	4821 independent reflections
Radiation source: fine-focus sealed tube	4137 reflections with *I* > 2σ(*I*)
graphite	*R*_int_ = 0.024
φ and ω scans	θ_max_ = 28.3°, θ_min_ = 1.7°
Absorption correction: multi-scan (*SADABS*; Bruker, 2005)	*h* = −8→8
*T*_min_ = 0.817, *T*_max_ = 0.942	*k* = −26→27
18749 measured reflections	*l* = −19→19

### Refinement

**Table:**

Refinement on *F*^2^	Primary atom site location: structure-invariant direct methods
Least-squares matrix: full	Secondary atom site location: difference Fourier map
*R*[*F*^2^ > 2σ(*F*^2^)] = 0.031	Hydrogen site location: inferred from neighbouring sites
*wR*(*F*^2^) = 0.081	H atoms treated by a mixture of independent and constrained refinement
*S* = 1.05	*w* = 1/[σ^2^(*F*_o_^2^) + (0.0382*P*)^2^ + 0.7829*P*] where *P* = (*F*_o_^2^ + 2*F*_c_^2^)/3
4821 reflections	(Δ/σ)_max_ < 0.001
251 parameters	Δρ_max_ = 0.40 e Å^−^^3^
3 restraints	Δρ_min_ = −0.30 e Å^−^^3^

### Special details

**Table:**

Geometry. All e.s.d.'s (except the e.s.d. in the dihedral angle between two l.s. planes) are estimated using the full covariance matrix. The cell e.s.d.'s are taken into account individually in the estimation of e.s.d.'s in distances, angles and torsion angles; correlations between e.s.d.'s in cell parameters are only used when they are defined by crystal symmetry. An approximate (isotropic) treatment of cell e.s.d.'s is used for estimating e.s.d.'s involving l.s. planes.
Refinement. Refinement of *F*^2^ against ALL reflections. The weighted *R*-factor *wR* and goodness of fit *S* are based on *F*^2^, conventional *R*-factors *R* are based on *F*, with *F* set to zero for negative *F*^2^. The threshold expression of *F*^2^ > σ(*F*^2^) is used only for calculating *R*-factors(gt) *etc*. and is not relevant to the choice of reflections for refinement. *R*-factors based on *F*^2^ are statistically about twice as large as those based on *F*, and *R*- factors based on ALL data will be even larger.

### Fractional atomic coordinates and isotropic or equivalent isotropic displacement parameters (Å^2^)

**Table:**

	*x*	*y*	*z*	*U*_iso_*/*U*_eq_	
Co1	0.5000	0.5000	0.5000	0.01335 (8)	
O1	0.54021 (15)	0.50614 (5)	0.36270 (6)	0.0169 (2)	
O2	0.87853 (15)	0.52883 (5)	0.37060 (7)	0.0196 (2)	
O3	0.76748 (16)	0.28543 (5)	0.75280 (7)	0.0219 (2)	
O4	0.20770 (15)	0.45517 (5)	0.45875 (7)	0.0182 (2)	
H41	0.162 (3)	0.4543 (8)	0.5144 (10)	0.023*	
H42	0.114 (3)	0.4789 (9)	0.4204 (11)	0.023*	
N1	0.63544 (17)	0.40399 (6)	0.51022 (8)	0.0155 (2)	
N2	1.09307 (19)	0.27891 (6)	0.71639 (8)	0.0191 (2)	
N3	0.6955 (2)	0.39432 (7)	−0.02756 (9)	0.0301 (3)	
C1	0.7089 (2)	0.50704 (6)	0.32932 (9)	0.0151 (3)	
C2	0.7037 (2)	0.47929 (7)	0.23444 (9)	0.0163 (3)	
C3	0.5191 (2)	0.45744 (7)	0.18361 (10)	0.0208 (3)	
H3	0.3938	0.4610	0.2092	0.025*	
C4	0.5143 (2)	0.43063 (8)	0.09649 (10)	0.0241 (3)	
H4	0.3857	0.4170	0.0628	0.029*	
C5	0.6975 (2)	0.42340 (7)	0.05741 (10)	0.0224 (3)	
C6	0.8826 (2)	0.44567 (8)	0.10906 (10)	0.0235 (3)	
H6	1.0089	0.4418	0.0844	0.028*	
C7	0.8845 (2)	0.47324 (7)	0.19537 (10)	0.0206 (3)	
H7	1.0118	0.4883	0.2286	0.025*	
C8	0.6312 (2)	0.36554 (7)	0.43557 (9)	0.0172 (3)	
H8	0.5692	0.3820	0.3774	0.021*	
C9	0.7131 (2)	0.30305 (7)	0.43964 (9)	0.0192 (3)	
H9	0.7071	0.2772	0.3855	0.023*	
C10	0.8040 (2)	0.27915 (7)	0.52449 (10)	0.0186 (3)	
H10	0.8615	0.2365	0.5295	0.022*	
C11	0.8101 (2)	0.31852 (7)	0.60242 (9)	0.0153 (3)	
C12	0.7227 (2)	0.38028 (7)	0.59202 (9)	0.0150 (3)	
H12	0.7248	0.4069	0.6452	0.018*	
C13	0.8903 (2)	0.29329 (6)	0.69730 (9)	0.0160 (3)	
C14	1.2464 (2)	0.29630 (8)	0.65634 (11)	0.0251 (3)	
H14A	1.3649	0.3187	0.6937	0.030*	
H14B	1.1825	0.3273	0.6086	0.030*	
C15	1.3266 (3)	0.23750 (10)	0.60918 (14)	0.0388 (4)	
H15A	1.4134	0.2523	0.5640	0.058*	
H15B	1.2091	0.2124	0.5776	0.058*	
H15C	1.4089	0.2099	0.6555	0.058*	
C16	1.1660 (2)	0.24446 (8)	0.80324 (11)	0.0248 (3)	
H16A	1.1023	0.2647	0.8537	0.030*	
H16B	1.3183	0.2495	0.8183	0.030*	
C17	1.1129 (3)	0.17229 (8)	0.79793 (12)	0.0311 (4)	
H17A	1.1568	0.1520	0.8582	0.047*	
H17B	1.1848	0.1513	0.7515	0.047*	
H17C	0.9628	0.1670	0.7806	0.047*	
C18	0.5005 (3)	0.37807 (9)	−0.08350 (11)	0.0334 (4)	
H18A	0.5267	0.3611	−0.1432	0.050*	
H18B	0.4139	0.4173	−0.0933	0.050*	
H18C	0.4287	0.3448	−0.0519	0.050*	
C19	0.8816 (3)	0.39299 (9)	−0.06991 (12)	0.0361 (4)	
H19A	0.8523	0.3719	−0.1305	0.054*	
H19B	0.9896	0.3685	−0.0305	0.054*	
H19C	0.9295	0.4378	−0.0775	0.054*	

### Atomic displacement parameters (Å^2^)

**Table:**

	*U*^11^	*U*^22^	*U*^33^	*U*^12^	*U*^13^	*U*^23^
Co1	0.01466 (13)	0.01570 (13)	0.00949 (12)	0.00226 (9)	0.00115 (9)	−0.00087 (9)
O1	0.0169 (5)	0.0216 (5)	0.0122 (4)	0.0031 (4)	0.0019 (4)	−0.0006 (4)
O2	0.0188 (5)	0.0258 (5)	0.0138 (5)	−0.0010 (4)	0.0015 (4)	−0.0012 (4)
O3	0.0251 (5)	0.0259 (5)	0.0155 (5)	0.0055 (4)	0.0056 (4)	0.0033 (4)
O4	0.0183 (5)	0.0221 (5)	0.0137 (5)	0.0017 (4)	0.0008 (4)	−0.0024 (4)
N1	0.0156 (5)	0.0179 (6)	0.0130 (5)	0.0010 (4)	0.0020 (4)	−0.0005 (4)
N2	0.0193 (6)	0.0192 (6)	0.0179 (6)	0.0021 (5)	0.0001 (5)	0.0018 (5)
N3	0.0372 (8)	0.0363 (8)	0.0173 (6)	0.0003 (6)	0.0056 (6)	−0.0098 (6)
C1	0.0194 (6)	0.0140 (6)	0.0118 (6)	0.0035 (5)	0.0016 (5)	0.0023 (5)
C2	0.0200 (7)	0.0168 (6)	0.0121 (6)	0.0016 (5)	0.0028 (5)	0.0014 (5)
C3	0.0207 (7)	0.0245 (7)	0.0180 (7)	−0.0003 (6)	0.0050 (6)	−0.0027 (6)
C4	0.0243 (8)	0.0290 (8)	0.0180 (7)	−0.0020 (6)	0.0000 (6)	−0.0041 (6)
C5	0.0319 (8)	0.0204 (7)	0.0153 (7)	0.0009 (6)	0.0052 (6)	−0.0018 (5)
C6	0.0248 (7)	0.0281 (8)	0.0193 (7)	0.0007 (6)	0.0091 (6)	−0.0032 (6)
C7	0.0205 (7)	0.0250 (7)	0.0164 (7)	0.0002 (6)	0.0034 (5)	−0.0010 (6)
C8	0.0185 (7)	0.0204 (7)	0.0126 (6)	−0.0010 (5)	0.0017 (5)	−0.0007 (5)
C9	0.0235 (7)	0.0194 (7)	0.0148 (6)	−0.0003 (5)	0.0035 (5)	−0.0048 (5)
C10	0.0213 (7)	0.0162 (6)	0.0186 (7)	0.0025 (5)	0.0038 (5)	−0.0021 (5)
C11	0.0150 (6)	0.0184 (6)	0.0128 (6)	0.0001 (5)	0.0030 (5)	0.0003 (5)
C12	0.0153 (6)	0.0171 (6)	0.0128 (6)	−0.0001 (5)	0.0022 (5)	−0.0019 (5)
C13	0.0204 (7)	0.0121 (6)	0.0150 (6)	0.0019 (5)	0.0008 (5)	−0.0017 (5)
C14	0.0182 (7)	0.0306 (8)	0.0264 (8)	−0.0030 (6)	0.0027 (6)	−0.0001 (6)
C15	0.0240 (8)	0.0493 (11)	0.0455 (11)	−0.0045 (8)	0.0126 (8)	−0.0178 (9)
C16	0.0257 (8)	0.0248 (8)	0.0210 (7)	0.0051 (6)	−0.0058 (6)	0.0027 (6)
C17	0.0355 (9)	0.0238 (8)	0.0331 (9)	0.0070 (7)	0.0016 (7)	0.0064 (7)
C18	0.0487 (11)	0.0339 (9)	0.0168 (7)	−0.0074 (8)	0.0026 (7)	−0.0067 (6)
C19	0.0560 (12)	0.0315 (9)	0.0251 (8)	−0.0078 (8)	0.0200 (8)	−0.0086 (7)

### Geometric parameters (Å, °)

**Table:**

Co1—O1	2.0701 (9)	C7—H7	0.9500
Co1—O1^i^	2.0701 (9)	C8—H8	0.9500
Co1—O4	2.1209 (10)	C9—C8	1.385 (2)
Co1—O4^i^	2.1209 (10)	C9—C10	1.385 (2)
Co1—N1	2.1519 (11)	C9—H9	0.9500
Co1—N1^i^	2.1519 (11)	C10—C11	1.3934 (18)
O1—C1	1.2676 (16)	C10—H10	0.9500
O2—C1	1.2611 (17)	C11—C12	1.3863 (19)
O3—C13	1.2332 (17)	C12—H12	0.9500
O4—H41	0.908 (13)	C13—C11	1.5026 (18)
O4—H42	0.907 (14)	C14—H14A	0.9900
N1—C8	1.3445 (17)	C14—H14B	0.9900
N1—C12	1.3393 (17)	C15—C14	1.519 (2)
N2—C13	1.3423 (18)	C15—H15A	0.9800
N2—C14	1.4696 (19)	C15—H15B	0.9800
N2—C16	1.4710 (18)	C15—H15C	0.9800
N3—C5	1.3782 (18)	C16—H16A	0.9900
N3—C18	1.446 (2)	C16—H16B	0.9900
N3—C19	1.442 (2)	C17—C16	1.518 (2)
C2—C1	1.4973 (18)	C17—H17A	0.9800
C3—C2	1.392 (2)	C17—H17B	0.9800
C3—H3	0.9500	C17—H17C	0.9800
C4—C3	1.385 (2)	C18—H18A	0.9800
C4—C5	1.406 (2)	C18—H18B	0.9800
C4—H4	0.9500	C18—H18C	0.9800
C6—C7	1.383 (2)	C19—H19A	0.9800
C6—C5	1.403 (2)	C19—H19B	0.9800
C6—H6	0.9500	C19—H19C	0.9800
C7—C2	1.3896 (19)		
			
O1^i^—Co1—O1	180.0	C8—C9—C10	118.46 (13)
O1—Co1—O4	89.24 (4)	C8—C9—H9	120.8
O1^i^—Co1—O4	90.76 (4)	C10—C9—H9	120.8
O1—Co1—O4^i^	90.76 (4)	C9—C10—C11	119.15 (13)
O1^i^—Co1—O4^i^	89.24 (4)	C9—C10—H10	120.4
O1—Co1—N1	90.78 (4)	C11—C10—H10	120.4
O1^i^—Co1—N1	89.22 (4)	C10—C11—C13	121.49 (12)
O1—Co1—N1^i^	89.22 (4)	C12—C11—C10	118.47 (12)
O1^i^—Co1—N1^i^	90.78 (4)	C12—C11—C13	119.79 (12)
O4—Co1—O4^i^	180.00 (5)	N1—C12—C11	122.85 (12)
O4—Co1—N1	88.07 (4)	N1—C12—H12	118.6
O4^i^—Co1—N1	91.93 (4)	C11—C12—H12	118.6
O4—Co1—N1^i^	91.93 (4)	O3—C13—N2	123.31 (13)
O4^i^—Co1—N1^i^	88.07 (4)	O3—C13—C11	118.90 (12)
N1—Co1—N1^i^	180.0	N2—C13—C11	117.76 (12)
C1—O1—Co1	128.10 (9)	N2—C14—C15	112.96 (14)
Co1—O4—H41	98.7 (11)	N2—C14—H14A	109.0
Co1—O4—H42	116.0 (11)	N2—C14—H14B	109.0
H42—O4—H41	106.6 (14)	C15—C14—H14A	109.0
C8—N1—Co1	121.32 (9)	C15—C14—H14B	109.0
C12—N1—Co1	120.62 (9)	H14A—C14—H14B	107.8
C12—N1—C8	118.05 (12)	C14—C15—H15A	109.5
C13—N2—C14	123.91 (12)	C14—C15—H15B	109.5
C13—N2—C16	117.90 (12)	C14—C15—H15C	109.5
C14—N2—C16	118.19 (12)	H15A—C15—H15B	109.5
C5—N3—C18	120.12 (14)	H15A—C15—H15C	109.5
C5—N3—C19	120.00 (14)	H15B—C15—H15C	109.5
C19—N3—C18	118.49 (13)	N2—C16—C17	112.39 (13)
O1—C1—C2	116.77 (12)	N2—C16—H16A	109.1
O2—C1—O1	124.70 (12)	N2—C16—H16B	109.1
O2—C1—C2	118.53 (12)	C17—C16—H16A	109.1
C3—C2—C1	121.19 (12)	C17—C16—H16B	109.1
C7—C2—C1	120.78 (13)	H16A—C16—H16B	107.9
C7—C2—C3	118.01 (13)	C16—C17—H17A	109.5
C2—C3—H3	119.3	C16—C17—H17B	109.5
C4—C3—C2	121.36 (14)	C16—C17—H17C	109.5
C4—C3—H3	119.3	H17A—C17—H17B	109.5
C3—C4—C5	120.77 (14)	H17A—C17—H17C	109.5
C3—C4—H4	119.6	H17B—C17—H17C	109.5
C5—C4—H4	119.6	N3—C18—H18A	109.5
N3—C5—C4	121.18 (14)	N3—C18—H18B	109.5
N3—C5—C6	121.37 (14)	N3—C18—H18C	109.5
C6—C5—C4	117.44 (13)	H18A—C18—H18B	109.5
C5—C6—H6	119.5	H18A—C18—H18C	109.5
C7—C6—C5	121.10 (14)	H18B—C18—H18C	109.5
C7—C6—H6	119.5	N3—C19—H19A	109.5
C2—C7—H7	119.3	N3—C19—H19B	109.5
C6—C7—C2	121.30 (14)	N3—C19—H19C	109.5
C6—C7—H7	119.4	H19A—C19—H19B	109.5
N1—C8—C9	123.02 (13)	H19A—C19—H19C	109.5
N1—C8—H8	118.5	H19B—C19—H19C	109.5
C9—C8—H8	118.5		

Symmetry codes: (i) −*x*+1, −*y*+1, −*z*+1.

### Hydrogen-bond geometry (Å, °)

**Table:**

*D*—H···*A*	*D*—H	H···*A*	*D*···*A*	*D*—H···*A*
O4—H41···O2^i^	0.91 (2)	1.78 (2)	2.6621 (14)	164 (2)
O4—H42···O2^ii^	0.91 (2)	1.90 (2)	2.7802 (14)	163 (1)
C9—H9···O3^iii^	0.95	2.41	3.3447 (17)	168
C19—H19A···O3^iv^	0.98	2.47	3.403 (2)	160
C15—H15A···Cg2^v^	0.98	2.86	3.734 (2)	148
C18—H18B···Cg1^vi^	0.98	2.86	3.7907 (19)	158

Symmetry codes: (i) −*x*+1, −*y*+1, −*z*+1; (ii) *x*−1, *y*, *z*; (iii) *x*, −*y*+1/2, *z*−1/2; (iv) *x*, *y*, *z*−1; (v) *x*+1, *y*, *z*; (vi) −*x*+1, −*y*+1, −*z*.
